# Mangiferin Attenuates Th1/Th2 Cytokine Imbalance in an Ovalbumin-Induced Asthmatic Mouse Model

**DOI:** 10.1371/journal.pone.0100394

**Published:** 2014-06-23

**Authors:** Hong-Wei Guo, Chen-Xia Yun, Guang-Han Hou, Jun Du, Xin Huang, Yi Lu, Evan T. Keller, Jian Zhang, Jia-Gang Deng

**Affiliations:** 1 Center for Translational Medicine, Guangxi Medical University, Nanning, Guangxi, China; 2 Key Laboratory of Longevity and Aging-related Disease, Chinese Ministry of Education, Nanning, Guangxi, China; 3 Guangxi Key Laboratory of Pharmacodynamic Studies of Traditional Chinese Medicine, Nanning, Guangxi, China; 4 School of Basic Medical Sciences, Guangxi University of Traditional Chinese Medicine, Nanning, Guangxi, China; 5 Department of Urology and Pathology, School of Medicine, University of Michigan, Ann Arbor, Michigan, United States of America; University of Pittsburgh Cancer Institute, United States of America

## Abstract

Mangiferin is a major bioactive ingredient in *Mangifera indica Linn.* (*Anacardiaceae*) leaves. Aqueous extract of such leaves have been used as an indigenous remedy for respiratory diseases like asthma and coughing in traditional Chinese medicine. However, underlying molecular mechanisms of mangiferin on anti-asthma remain unclear. In our present study, we investigated the anti-asthmatic effect of mangiferin on Th1/Th2 cytokine profiles and explored its underlying immunoregulatory mechanism in mouse model of allergic asthma. Mangiferin significantly reduced the total inflammatory cell counts and eosinophil infiltration, decreased the production of ovalbumin-specific IgE in serum and PGD_2_ in BALF. The antibody array analysis showed that mangiferin down-regulated the levels of one group of cytokines/chemokines including Th2-related IL-4, IL-5, IL-13, and others IL-3, IL-9, IL-17, RANTES, TNF-α, but simultaneously up-regulated Th1-related IFN-γ, IL-2 and IL-10 and IL-12 expression in serum. Thus it attenuates the imbalance of Th1/Th2 cells ratio by diminishing the abnormal mRNA levels of Th1 cytokines (IFN-γ and IL-12) and Th2 cytokines (IL-4, IL-5 and IL-13). Finally, mangiferin substantially inhibited the activation and expression of STAT-6 and GATA-3 in excised lung tissues. Our results suggest that mangiferin can exert anti-asthmatic effect. The underlying mechanism may attribute to the modulation of Th1/Th2 cytokine imbalance via inhibiting the STAT6 signaling pathway.

## Introduction

Allergic asthma is a chronic inflammatory disease of the bronchial airways characterized by infiltrating of a variety of inflammatory cells, including eosinophils, mast cells, T-lymphocytes, neutrophils, and macrophages among others [1]. In recent years, the incidence and severity of atopic disorders has steadily increased in developed countries [2]. It has been reported that allergic asthma is tightly associated with imbalance of Th1/Th2 cells and their characteristic cytokine profiles [3]. Th2 cell responses initiate and predominate in atopic disorders through releasing of Th2 cytokines, mainly IL-4, IL-5 and IL-13, which elevate the serum immunoglobulin E and recruit eosinophils to airways, thus further inducing secretion of histamine, leukotriene and prostaglandin. Compared with Th2 cells, Th1 cytokines, such as IFN-γ and IL-12, are involved in antagonism of Th2 cell responses and IgE synthesis to restrain the progress of asthma. In physiological condition, immune responses of Th1 and Th2 cells maintain dynamic balance. Whenever this balance is disturbed, diseases will occur. Therefore, one effective treatment for asthma is to try to improve Th1 immune responses and simultaneously inhibit Th2 immune responses to restore Th1/Th2 balance [4].

Signal transducer and activator of transcription (STAT) proteins are a group of transcription factors that transmit signals from extracellular milieu of cells to nucleus. It has been demonstrated that the activation of STAT4 and STAT6 is pivotal in naive CD4+ T (Th0) cell differentiate along Th1 and Th2 pathways [5]. Previous studies indicated that immunization of STAT4-deficient mice resulted in a typical Th2-like immune response [6]. In addition, protein content and mRNA levels of STAT6 in asthma patients/models appeared to arise, STAT4 decreased abnormally [7], [8]. T-box expressed in T-cells (T-bet), a key transcription factor of Th1 cells, has been identified to promote Th1 development and IFN-γ production [9]. What's more, IFN-γ has been shown to induce T-bet expression, which results in a potential positive feedback loop during Th1 cell differentiation [10]. GATA-binding protein-3 (GATA-3), as a downstream transcriptional factor of STAT6, plays an important role in Th2 cell development by promoting Th2 cytokine expression through binding to a variety of regulatory regions of Th2 cytokines [11], [12]. At the same time, GATA-3 induction inhibits Th1 differentiation both by increasing IL-4 production, and by inhibiting the master Th1 transcription factor T-bet [13]. Thus, biological compounds targeting these molecules may provide an effective therapeutic modality for patients with asthma.

**Figure 1:**
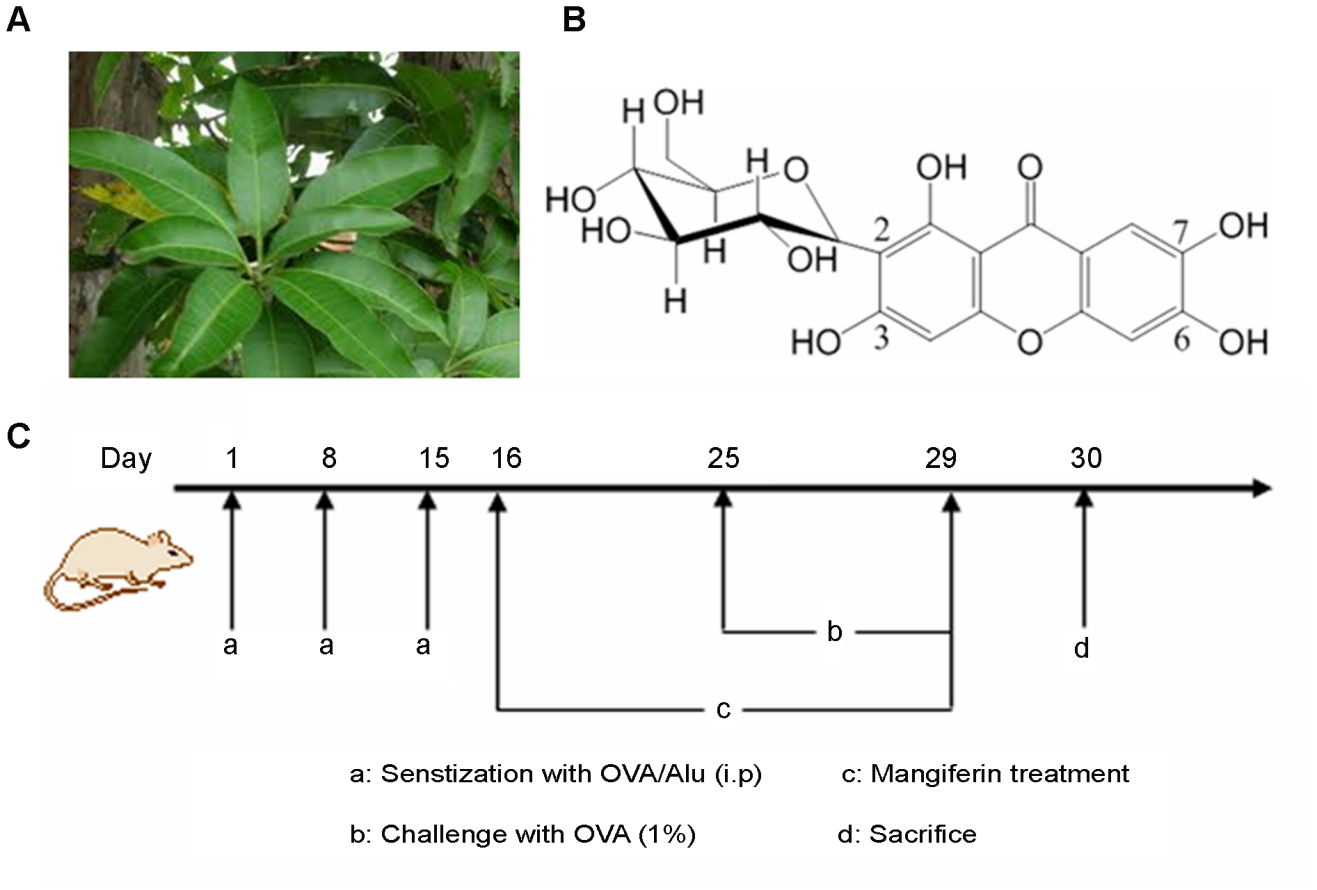
Experimental drugs and treatment schedule. (A) The leaves of *Mangifera indica Linn* (B) Chemical structure of mangiferin (C) Sensitization, challenge and treatment schedule in this study.

Mangiferin, a natural C-glucoside xanthone (1,3,6,7-Tetrahydroxy-2-[3,4,5-trihydroxy-6-(hydroxymethyl)oxan-2-yl]xanthen-9-one) and its PubChem identification number (CID 5281647), is abundantly present in *Mangifera indica Linn* leaves. (Figure 1A and 1B) [14]. Aqueous extracts of such leaves have been traditionally used for decades as a remedy for respiratory diseases in Chinese medicine [15]. In Cuba, Vimang products are used for treating inflammatory diseases and cancer [16], [17]. Previous published pharmacological studies, both in vitro and in vivo, indicate that mangiferin has pleiotropic bioactivities, including anti-oxidant [18], [19], anti-tumor [20], anti-microbial [21], anti-diabetic [22], [23], hepato-, cardio- and radioprotective [24], [25], [26], [27], and anti-allergic [28] activities. Besides, it also exhibits anti-inflammatory [29] and immunomodulatory [30], [31] properties. There has not yet been found any clinical evidence of adverse effects of mangiferin. Therefore, it could become a promising candidate for developing natural medicine [32].

In our previous studies, we reported that mangiferin had beneficial effects on treatment of chronic inflammation and chronic bronchitis [15], [29]. Recently, anti-asthmatic properties of mangiferin have also been initially described by Rivera DG (2011) [33]. They reported that mangiferin could inhibit the airway inflammation based on the histological changes and the reduction of IL-4, IL-5 and IgE levels. However, underlying molecular mechanisms of mangiferin, such as the contributions of Th1 vs Th2 responses, on anti-asthma remain unclear. In our prior reports, we have not determined the molecular mechanisms.

## Materials and Methods

### Ethics Statement

Experimental conditions and procedures involving animals were approved by Institutional Animal Ethics Committee (IAEC), Guangxi University of Traditional Chinese Medicine, and carried out in accordance with laboratory animal use guidelines of IAEC (Permit Number: SCXK (GUI) 2009-0002). Animal handling followed the National Animal Welfare Law of China.

### Reagents

Chicken egg ovoalbumin (OVA), Aluminum hydroxide Gel (13 mg/ml) were purchased from Sigma (St Louis, USA). Quantibody Mouse Cytokine Array 1 kit was purchased from RayBiotech Inc. (Norcross, USA), ELISA kits were purchased from R&D (Minneapolis, MN, USA). Antibodies for anti-CD_3_ (FITC), anti-CD_4_ (PE-Cy7), anti-IL-4 (PE), anti-IFN-γ (APC), PE-Rat IgG1, APC-Rat IgG1, κ isotype control were purchased from BD Biosciences (Franklin Lakes, New Jersey, USA). Anti-STAT4, STAT6, phosphorylated STAT4, phosphorylated STAT6, GAPDH, antibodies were purchased from Cell Signaling Technology (Danvers, USA). All other chemicals and reagents were of analytical reagent grade purchased from Sigma. Mangiferin (98.39%, HPLC), has been used elsewhere [29], were obtained from Guangxi Key Laboratory of Pharmacodynamic Studies of Traditional Chinese Medicine (Nanning, China). Dexamethasone was purchased from Zhejiang Xianju Pharmaceutical co., LTD (Hangzhou, China),

### Animals

Specific pathogen-free female Balb/c mice, 6–8 weeks old, 18±2 g, were purchased from the Guangxi Medical University Laboratory Animal Center (Nanning, China). They were housed at Guangxi University of Traditional Chinese Medicine in polypropylene cages in a standard bio-clean animal room, and kept under a 12 h light–dark cycle at 25±2°C. The mice were given food and water available ad libitum, and were allowed to acclimatize for one week before the experiments.

### Sensitization, challenge and treatment

Sensitization and challenge were performed as described by Ram A (2004), with some modifications [34]. Mice were arbitrarily assigned into six groups and each consisted of 12 mice, namely normal group, model group, magiferin treated groups (with 50 mg/kg, 100 mg/kg, and 200 mg/kg), and dexamethasone group.

Animal in model group, magiferin treated groups and dexamethsone group, were intraperitoneally (i.p) sensitized with 20 µg of OVA and 0.15 ml aluminum hydroxide gel in 0.2 ml phosphate buffer saline (PBS, pH 7.4). Injections were given three times on Day 1, 8 and 15. The sensitized mice were individually placed in a in a plexiglass chamber and challenged by repeated exposure to an aerosol of OVA (1%), which was delivered by a PARI BOY N (085G1205) nebulizer (PARI GmbH, Starnberg, German) driven by compressed air at 20 L/min. Challenge was done for 20 min once a day for 5 consecutive days (Day 25–29). The normal group were received intraperitoneal injections of 0.2 ml PBS (pH 7.4) containing 0.15 ml aluminum hydroxide gel and were challenged with PBS alone.

To evaluate the protective effect, mice were orally treated with mangiferin (50 mg/kg, 100 mg/kg, and 200 mg/kg) from Day 16 to 29 (14 consecutive days). Saline was used as vehicle and the mangiferin was administered once a day. Dexamethasone (1.25 mg/kg), used as reference drug for positive control [33], was orally administered from Day 16 to 29. Normal group and model group were received only saline. Animals were sacrificed 24 h after the last challenge (thus on Day 30) to investigate the suppressive effects of mangiferin. The treatment schedule is shown in Figure 1C.

### Collection of bronchoalveolar lavage fluid (BALF) and blood

Twenty four hours after the last challenge, the mice were bled via the retroorbital plexus and sera were separated by centrifugation (3000 rpm, 10 min at 4°C) and kept at −80°C until analysis for cytokines and OVA-specific IgE. Then, the mice were sacrificed using sodium pentothal (50 mg/kg, i.p.), and tracheotomy was performed. The trachea was cannulated and 0.6 ml of ice-cold PBS was used to instill into a lung for lavage. This process was repeated once for each mouse. About 1 ml of BALF was obtained per mouse. The BALF was centrifuged (3000 rpm, 10 min at 4°C) and the supernatants were kept at −80°C until analysis for leukotrienes C_4_ (LTC_4_) and prostaglandin D_2_ (PGD_2_). The pellet was resuspended in 300 µl of ice-cold PBS, centrifuged onto slides and stained for 5 min with Wright–Giemsa staining. The slides were quantified for differential cell count by counting a total of 300 cells/slide at 40 magnification. The total number of BALF cells was counted using a haemocytometer.

### Histological assessment

Histological assessment of inflammatory changes in lung tissues was made in each animal. The lung tissues were excised intact and then fixed overnight by perfusing with 4% paraformaldehyde and subsequently embedded in paraffin. Samples were removed from the middle, cranial, accessory and caudal lobes. A transverse section of 4 µm thick was cut from each sample and stained with haematoxylin and eosin (H&E). Slides were evaluated by microscopy and inflammatory changes were scored in a double-blind manner with two independent researchers. Underwood et al. (1995) noted that the degree of peribronchial and perivascular inflammation was evaluated by a subjective scale of 0–5 points, as described in Table 1 [35].

**Table 1:** Histopathological scoring system used to assess inflammatory change in the lungs.

Histopatho-logy grade	Perivascular and peribronchiolar eosinophilia	Oedema	Epithelial damage
0	Normal	Normal	Normal
1	Low grade cell influx, no tissue pathology	Low grade diffuse oedema	Low grade cell loss
2	Low to moderate cell influx, low grade tissue damage	Moderate alveolar and bronchiolar oedema	Low grade cell loss
3	Moderate cell influx, low grade tissue damage	Regional and focal oedema	Moderate cell loss
4	Moderate to high cell influx, marked tissue damage	Pronounced oedema	Moderate cell loss
5	High cell influx, significant tisssue pathology	Pneumonic-type oedema	Epithelial metaplasia, mucus cell hyperplasia

### Detection of LTC_4_ and PGD_2_ in BALF by ELISA

BALF levels of LTC_4_ and PGD_2_ were measured using ELISA according to the manufacturer's instructions. All measurements were performed in duplicate. Briefly, the serum samples were added in duplicate to 96-well plates with 100 ml per well. The appropriate biotinconjugated antibodies were added to each well. The samples were incubated at room temperature for 2 h. The wells were then aspirated, and each well was washed 5 times. The substrate solutions were added to each well, and were incubated for 30 min at room temperature in the dark. The optical density (OD) of each well was determined using a microplate reader (Bio-Rad Model 680, USA) that was set to 450 nm. A standard curve was created of the average of the OD duplicate readings.

### Measurement of OVA-specific IgE levels in serum

OVA-specific IgE levels in serum were analyzed by ELISA as described previously [36], using OVA to capture the antibodies, sheep anti-mouse IgE as the secondary antibody, and HRP-conjugated rabbit anti-sheep IgG as the tertiary antibody. The samples were diluted 100 times in appropriate buffer and were individually evaluated in duplicate. The results are shown as absorbance units at 492 nm.

### Antibody array detection of cytokines levels in serum

All cytokines levels in the serum samples were measured using Quantibody mouse cytokine array kit (QAM-CYT-1-1, RayBiotech, Inc., Norcross, USA) in which 20 cytokines/chemokines were quantitatively detected: interleukin (IL)-1α, IL-1β, IL-2, IL-3, IL-4, IL-5, IL-6, IL-9, IL-10, IL-12, IL-13, IL-17, keratinocyte-derived chemokine (KC), monocyte chemoattractant protein-1(MCP-1), macrophage colony stimulating factor (M-CSF), regulated upon activation, normal T expressed and secreted (RANTES), tumor necrosis factor alpha (TNF-α), vascular endothelial growth factor (VEGF), granulocyte-macrophage colony stimulating factor (GM-CSF), and interferon-gamma (IFN-γ). The assay was performed according to the manufacturer's protocol and data were analyzed with the software provided by the company.

### Semi-quantitative RT-PCR

Total RNA was extracted from homogenized lung tissues using RNAprep pure Tissue Kit (TIANGEN Biotech Co., Ltd, Beijing, China) and converted to cDNA using oligo-dT and random primers. PCR amplification (Taq PCR Mastermix, TIANGEN Biotech Co., Ltd, Beijing, China) was performed on a 2 µl-sample of cDNA. Amplification cycle numbers and annealing temperatures were optimized for each primer pair. PCR products were electrophoresed on 1.5% agarose gel and stained with ethidium bromide. Oligonucleotide sequences of PCR primers for IL-4, IL-5, IL-12, IL-13, IFN-γ and β-actin are listed in Table 2.

**Table 2:** Oligonucleotide sequences of Semi-quantitative RT-PCR.

Gene	Primer	Sequences(5′ to 3′)	Amplified fragment size
β-actin	Forward	TGGAATCCTGTGGCATCCATGAAAC	349bp
	Reverse	TAAAACGCAGCTCAGTAACAGTCCG	
IL-4	Forward	CCTGCTCTTCTTTCTCGAATGT	165bp
	Reverse	CTCTCTGTGGTGTTCTTCGTTG	
IL-5	Forward	TCAGCTGTGTCTGGGCCACT	133bp
	Reverse	TTATGAGTAGGGACAGGAAGCCTCA	
IL-12α	Forward	ATTATTCCTGCACTGCTGAAGAC	394bp
	Reverse	TTCACTCTGTAAGGGTCTGCTTC	
IL-13	Forward	TGACCAACATCTCCAATTGCA	132bp
	Reverse	TTGTTATAAAGTGGGCTACTTCGATTT	
IFN-γ	Forward	GGGACAGCCAAGCGGCTGAC	98bp
	Reverse	CACCTCCCGGGGTCACTGCA	

### Flow cytometric analysis of Th1 and Th2 cells

The spleen was aseptically removed from each sacrificed mouse and placed in a tube containing RPMI1640 media. A single cell suspension was prepared by disrupting the spleen using Cell disrupter (Beckman) and then cells were equally distributed into 96-well plate and incubated with PMA (50 ng/ml) and ionomycin (1 µg/ml) for 5 h in a 5% CO_2_ humidified incubator. Then cells were labeled with anti-mouse CD3 and CD4 antibody (BD, USA) at 4°C for 30 min. Following surface staining, cells were fixed and permeabilized in Perm/Wash buffer for 20 min at 4°C in the dark, then incubated with anti-IL-4 antibody, anti-IFN-γ antibody (BD Biosciences, USA) for 30min at 4°C in the dark. After fixed in 1% paraformaldehyde, the stained cells were detected by flow cytometer (Beckman Colter, USA).

### Immunohistochemistry (IHC) analysis of STAT4 and STAT6

For immunohistochemistry analysis, the paraffin-embedded sections of lung tissues were deparaffinized with xylene and then rehydrated. Section slides were incubated with 3% hydrogen peroxide for 10min and then in 5% BSA in PBS blocking solution for 20min, and incubated with anti-STAT4 or anti-STAT6 antibody (Cell signaling Technology) in blocking solution overnight at 4°C. After washing with PBS, the slides were treated with biotinylated secondary antibody for 20 min, streptavidin-HRP (horseradish peroxidase) for 20 min, and 3,3N-Diaminobenzidine Tertrahydrochloride for 10 min (DAKO, Carpinteria, CA). The slides were then washed and counter stained with hematoxylin. Slides were evaluated by microscopy and the positive cells showed yellow or brown particles or clumps in the cytoplasm with a blue colored cellular nucleus.

### The protein levels of p-STAT4/6, GATA3 and T-bet by western blot

The expressions of phosphorylated STAT4 (p-STAT4),phosphorylated STAT6 (p-STAT6), GATA3 and T-bet in lung tissues were detected by western blot. We homogenized the lung tissues, washed them with PBS, and incubated them in lysis buffer in the presence of protease inhibitor cocktail (Sigma, St Louis, USA) to obtain extracts of lung proteins. The samples were loaded to 10% SDS-PAGE gels and were transferred to polyvinylidene fluoride membranes. The blots were incubated with the appropriate concentration of specific primary antibody overnight at 4°C. After washing, the blots were incubated with peroxidase-conjugated second antibody. The membranes were stripped and reblotted with GAPHD antibody (CST, Danvers, USA) or α-tubulin antibody (Sigma, St Louis, USA) to verify the equal loading of protein in each lane.

### Statistical analysis

Data were expressed as mean ± SD and analyzed by one-way ANOVA followed by LSD and Dunnett's T3 using SPSS version 11.5 software. *P*<0.05 was considered as statistical significance.

## Results

### Mangiferin decreased the OVA-induced eosinophilia and BALF composition

**Figure 2:**
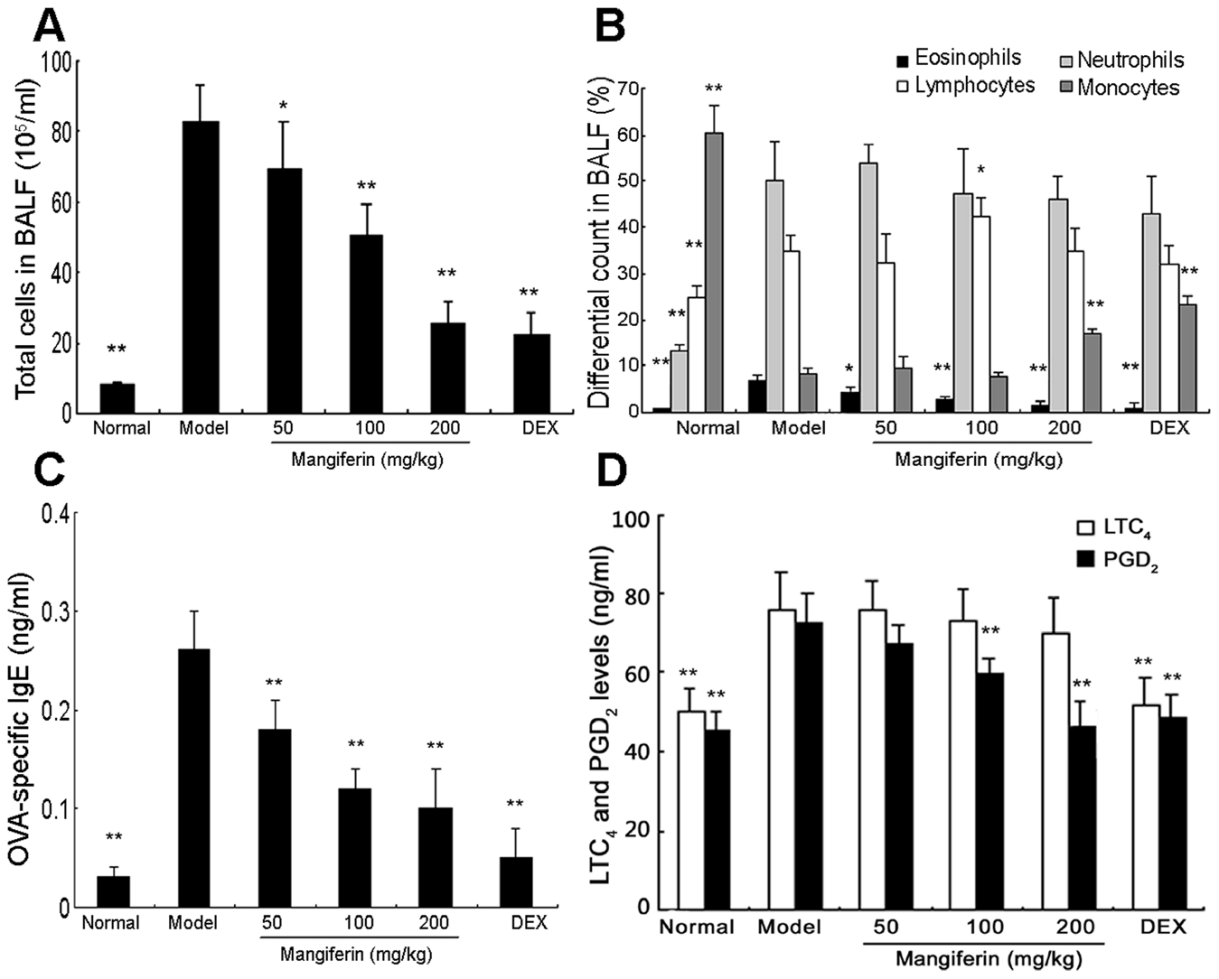
Effects of mangiferin on OVA-induced eosinophil infiltration, BALF composition, and levels of IgE, LTC_4_ and PGD_2_ in experimental mice. BALF and serum were collected at 24h after the last OVA challenge. The total cells and differential cells in BALF were counted as described in Materials and Methods. The levels of OVA-specific IgE in serum, LTC_4_ and PGD_2_ in BALF were measured by ELISA (C and D). The data are representative of three independent experiments and represented as the means ± SDs (n = 12). Compared with model group: ^*^ *P*<0.05; ^**^ *P*<0.01.

Eosinophil plays a key role in allergic diseases such as asthma [37]. To determine the effect of mangiferin on airway inflammation, we counted both total cell and differential cells including eosinophils, neutrophils, lymphocytes and monocytes in BALF. Compared with the normal group, OVA induced a marked influx of leukocytes into BALF and the ratio of eosinophil in leukocytes markedly increased (Figure 2A and 2B). OVA-sensitized and-challenged mice treated with mangiferin displayed significantly reduced leukocytosis and a decreased ratio of eosinophilia. These reductions by mangiferin were in a dose-dependent manner, and comparable with the dexamethasone treatment. Dexamethasone was included as a positive control for anti-inflammatory effects, and would be included in other experiments as well to show the similar effectiveness or better results (in some cases) by mangiferin.

### Mangiferin inhibited the release of IgE, LTC_4_ and PGD_2_

Airway inflammation is associated with higher levels of allergen-specific IgE and inflammatory mediators such as LTC_4_ and PGD_2_ [38]. In this study, levels of OVA-specific IgE in serum, LTC_4_ and PGD_2_ in BALF were measured by ELISA 24 hours after the last OVA challenge. An abnormal increase in OVA-specific IgE as well as LTC_4_ and PGD_2_ in OVA-treated mice (model group) was observed. In contrast, mangiferin treatment dramatically reduced the levels of OVA-specific IgE in serum in a dose-dependent manner (Figure 2C). Additionally, PGD_2_ expression in BALF were markedly suppressed by mangiferin at 100 and 200 mg/kg as shown in Figure 2D. However, the expression of LTC_4_ was slightly suppressed by mangiferin but did not show statistical differences.

### Mangiferin diminished the OVA-induced eosinophilia in lung tissues

**Figure 3:**
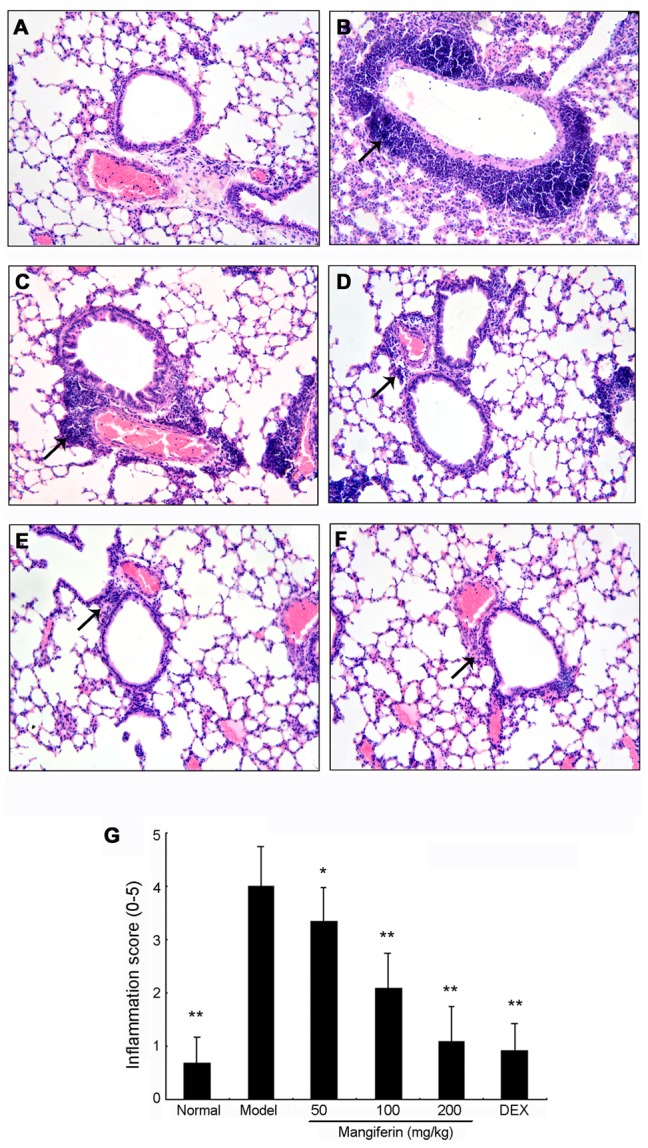
Effects of mangiferin on OVA-induced lung histological changes in experimental mice. (A) Normal; (B) Model; (C) Mangiferin 50 mg/kg, (D) Mangiferin 100 mg/kg, (E) Mangiferin 200 mg/kg, (F) Dexamethasone 1.25 mg/kg, (G) Qualitative analysis of anti-inflammatory effects of mangiferin based on inflammation scores as described in Materials and Methods. H&E staining, magnification 200×, only a representative picture is shown for each group. Twelve mice have been used in each group and all *in vivo* experiments were repeated for three times. Data are expressed as means ± SDs. Compared with model group: ^*^ *P*<0.05; ^**^ *P*<0.01.

Given that mangiferin inhibits inflammatory cells recruitment into the BALF, we evaluated if mangiferin can impose the anti-inflammatory effects on lung tissues by H&E staining. Few inflammatory cells were found in the lungs of normal group (Figure 3A). However, in OVA-induced asthmatic lung tissue (Figure 3B), we observed significant infiltration of inflammatory cells into peribronchial and perivascular connective tissues, compared with the normal tissues. The infiltrating cells, in the inflamed tissue, seemed to be leukocytes based on morphology under microscope. Moreover, most leukocytes were eosinophils. In the mice with mangiferin and dexamethasone treatment, we found that infiltration of eosinophil-rich leukocytes was significantly attenuated compared to what was observed in the model group as shown in Figure 3C-3F. In addition, the inflammation score in model group was around 4, indicating a moderate to high level infiltration by eosinophil. This score was reduced when the mice were treated with mangiferin. The highest dose of the mangiferin (200 mg/kg) had similar effect to dexamethasone. (Figure 3G).

### Mangiferin had reciprocal effects on Th1 and Th2 related cytokines/chemokines secretion

**Figure 4:**
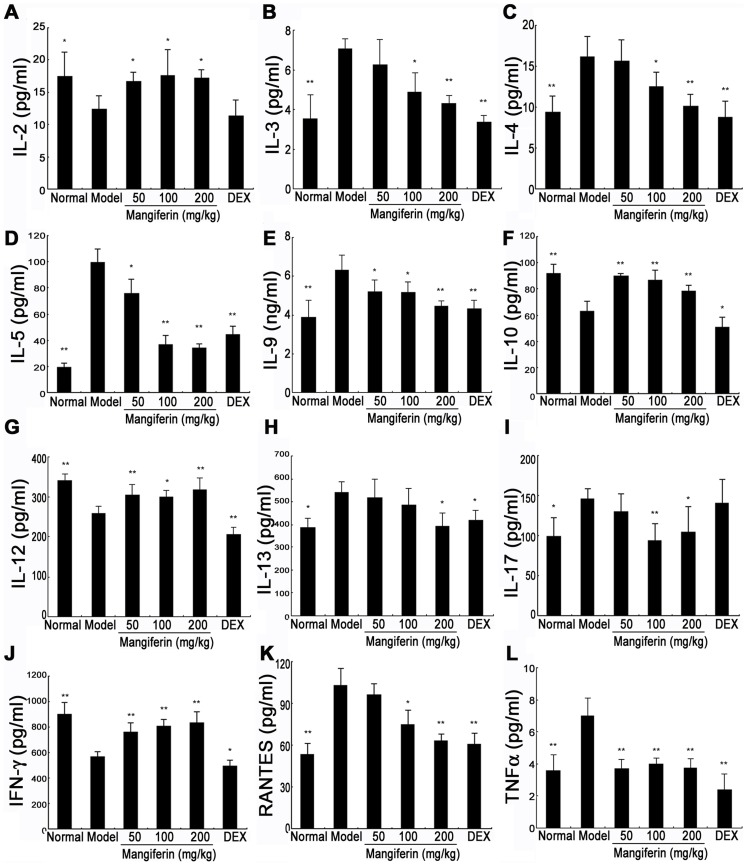
Effect of mangiferin on inflammatory cytokines/chemokines in serum of experimental mice. The levels of 20 cytokines/chemokines in serum samples were quantitatively detected by Quantibody mouse cytokine array kit and indicated in each panel. The data are representative of three independent experiments and represented as the means ± SDs (n = 12). Compared with model group: ^*^ *P*<0.05; ^**^ *P*<0.01.

Inflammatory cytokines, such as Th2 cytokines (including IL-4, IL-5, and IL-13), Th1 cytokines (including IFN-γ, IL-2 and IL-12), proinflammatory cytokines (IL-1, IL-6 and TNF-α), IL-9, IL-10, IL-17 and chemokines (including RANTES and MCP-1) contribute to the pathogenesis of chronic allergic asthma [39], [40], [41]. To investigate the underlying immunoregulatory mechanism of mangiferin on anti-asthma, the serum levels of cytokines and chemokines associated with airway inflammation were detected by antibody array. As shown in Figure 4., in model group mice, several cytokine/chemokine levels, including IL-3, IL-4, IL-5, IL-9, IL-13, IL-17, RANTES and TNF-α in the serum, were higher than those in normal group mice, while the levels of IFN-γ, IL-2, IL-10 and IL-12 was significantly decreased. Under this condition, mangiferin treatment markedly attenuated the increase in the level of IL-3, IL-4, IL-5, IL-9, IL-13, IL-17, RANTES and TNF-α. Interestingly, mangiferin also elevated the expression of IFN-γ, IL-2, IL-10 and IL-12. However, Mangiferin did not affect the expressions of other cytokines and chemkines such as GM-CSF, IL-1α, IL-1β, IL-6, MCP-1, KC, M-CSF and VEGF (data not shown).

### Mangiferin attenuated imbalanced mRNAs levels of Th1 and Th2 cytokines

**Figure 5:**
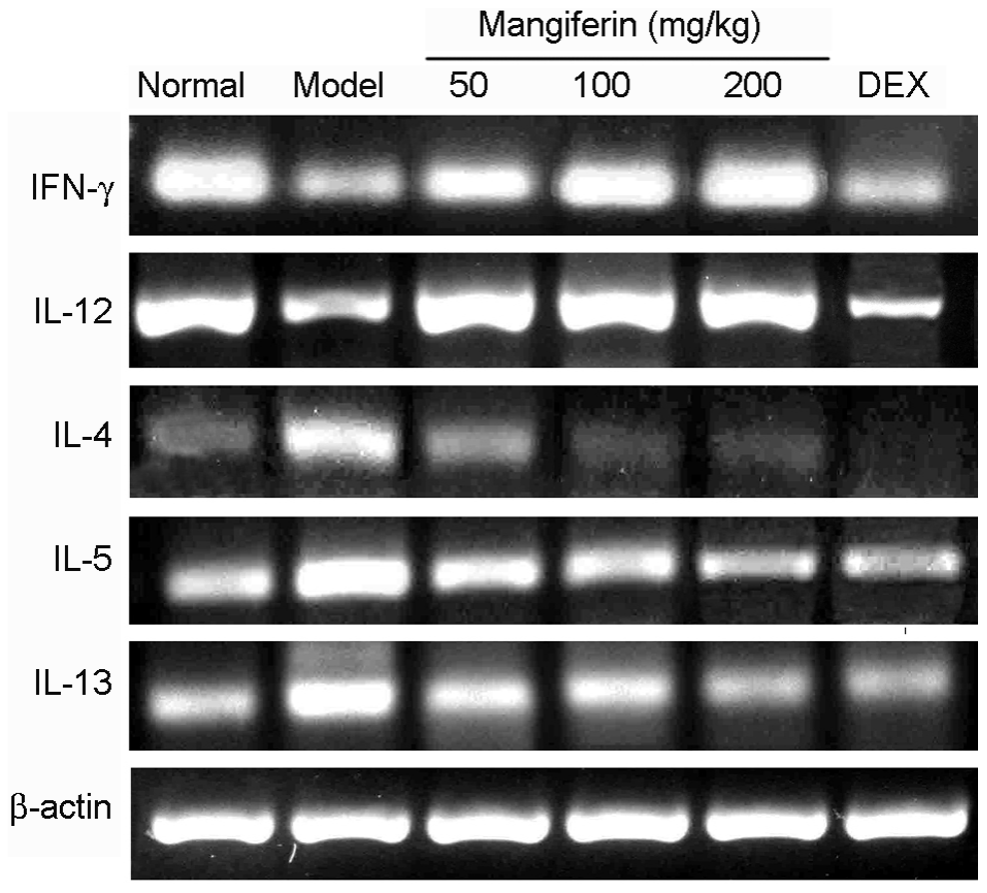
Effect of mangiferin on mRNA levels of Th1 and Th2 cytokines in the lung tissues. Total RNA was extracted from each lung, and the mRNA expression of Th1 cytokines (IFN-γ, IL-12) and Th2 cytokines (IL-4, IL-5 and IL-13) were determined by RT-PCR as described in Materials and Methods. β-actin was used as an internal control for each PCR reaction. Three independent experiments were examined (12 mice in each group of one experiment) and a representative RT-PCR profile was shown.

As antibody array showed that mangiferin suppressed characteristic cytokines secreted by Th1/Th2 cells such as IFN-γ, IL-12 and IL-4, IL-5, IL-13, which played the key roles in asthma. To confirm this, IFN-γ, IL-12, IL-4, IL-5, and IL-13 mRNAs from lung tissues were amplified and determined by Semi-quantitative RT-PCR. As expected, treatment with mangiferin, mRNA expressions of IFN-γ and IL-12 were significantly increased and mRNA levels of IL-4, IL-5 and IL-13 were markedly suppressed, especially in high dose group (Figure 5.). These results confirmed that imbalance of Th1/Th2 cytokines could be attenuated by mangiferin.

### Mangiferin restored the ratio of Th1 and Th2 cells within CD4 populations

**Figure 6:**
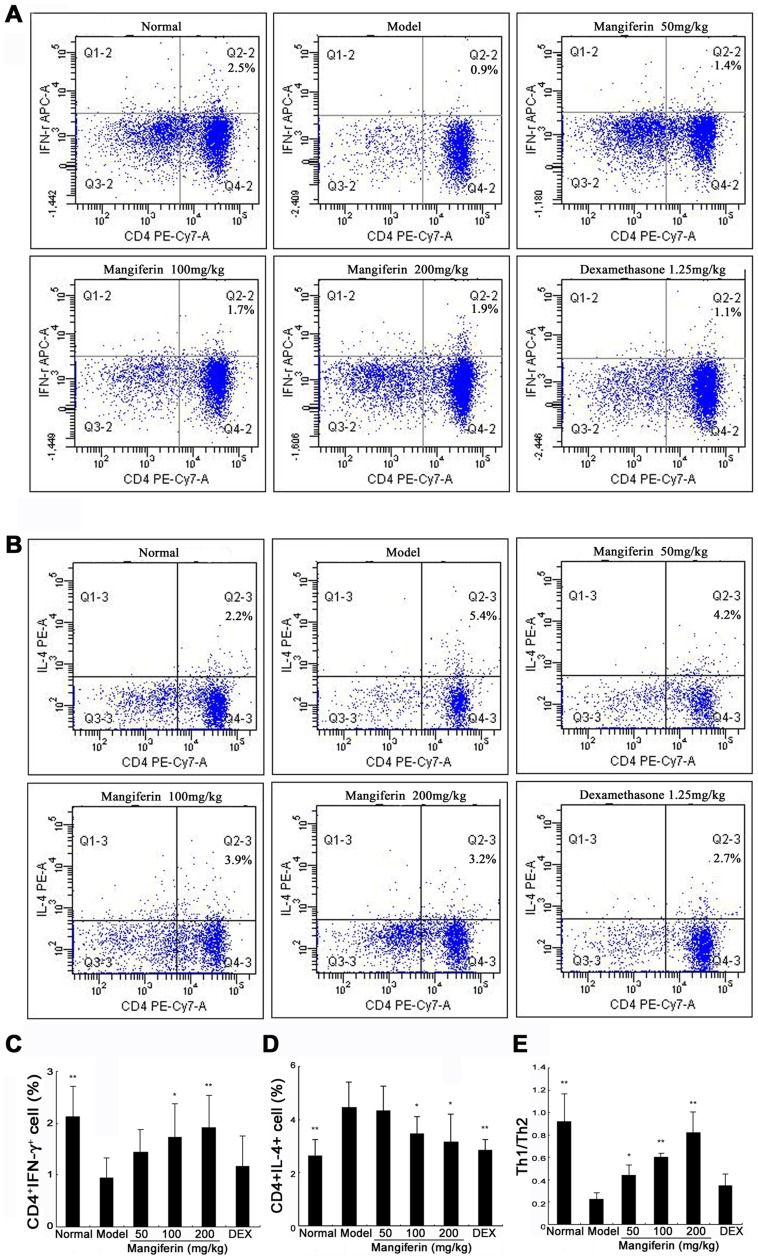
Effect of mangiferin on percentages of Th1 and Th2 cells among CD4^+^ T cells in spleens of experimental mice. (A) Flow cytometric analysis of splenocytes stained successively with Anti-CD3 (FITC), Anti-CD4 (PE-Cy7) and anti-IFN-γ (APC). (B) Flow cytometric analysis of splenocytes stained successively with Anti-CD3 (FITC), Anti-CD4 (PE-Cy7) and anti-IL-4 (PE). (C) Bar plot of the average percentage of CD4^+^ IFN-γ^+^ cells. (D) Bar plot of the average percentage of CD4^+^ IL-4^+^ cells. (E) The ratio of CD4^+^ IFN-γ^+^ (Th1) cell and CD4^+^ IL-4^+^ (Th2) cell. Twelve mice have been used in each group and all *in vivo* experiments were repeated for three times. Data are expressed as means ± SDs. Compared with model group: *P<0.05; **P<0.01.

It has been suggested that Th1 and Th2 cells played a major role when asthma occurs. For example, imbalanced status of Th1/Th2 cells is found in bronchial asthma. A series of allergic reaction could occur when either Th1 cell activity was reduced or Th2 cell activity was enhanced [42]. In current study, percentages of CD4^+^ IL-4^+^ T (Th2) cells in the model group were higher than those in the normal group, in contrast, percentages of CD4^+^ IFN-γ^+^ T (Th1) cells decreased significantly. Compared with model group, mangiferin reduced frequencies of Th2 cells and increased frequencies of Th1 cells in a dose-dependent manner, with the high dose group demonstrating the most effective (Figure 6A-6D). Furthermore, Th1/Th2 ratio significantly increased in treatment groups. Treatment with mangiferin showed restoration of Th1/Th2 ratio (Figure 6E).

### Inhibiting the activation and expression of STAT-6 and GATA-3 may be the underlying mechanism by mangiferin

STAT-4, STAT-6, T-bet and GATA-3 have been reported to play a critical role during the native CD4^+^ T (Th0) cells differentiating to Th1 and Th2 cells [5], [43]. Therefore, we examined whether the activation and protein expression of STAT-4, STAT-6, GATA-3 and T-bet would be regulated by mangiferin using IHC and western blot, respectively.

**Figure 7:**
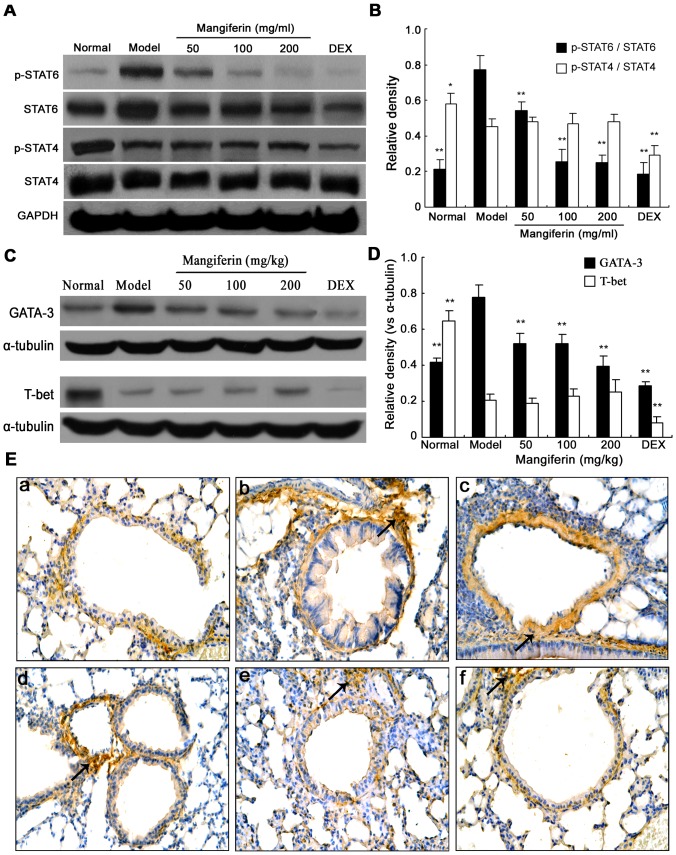
Effects of mangiferin on the protein expressions and phosphorylation levels of STAT4 and STAT6 in lung tissues of experimental mice. (A) The levels of p-STAT6 and p-STAT4 in lung tissues were measured by western blot. (B) Bar plot of the average relative density of p-STAT4 and p-STAT6. (C) The levels of GATA3 and T-bet in lung tissues were measured by western blot. (D) Bar plot of the average relative density of GATA3 and T-bet. (E) The expression of STAT6 in lung tissues was detected by immunohistochemistry (magnification 400×), only a representative picture is shown for each group. (a) Normal; (b) Model; (c) Mangiferin 50 mg/kg, (d) Mangiferin 100 mg/kg, (e) Mangiferin 200 mg/kg, (f) Dexamethasone 1.25 mg/kg. Three independent experiments were examined (12 mice in each group of one experiment), Data are expressed as means ± SDs. Compared with model group: ^*^ *P*<0.05; ^**^ *P*<0.01.

Western blot revealed that the level of p-STAT6 and GATA-3 in the model group increased significantly compared with the normal group, while the level of p-STAT4 and T-bet significantly decreased. Mangiferin inhibited increased levels of p-STAT6 and GATA-3 in mangiferin treated groups, but not the level of p-STAT4 and T-bet (Figure 7A-7D). In addition, IHC analyses revealed similar results as Western blot. Consistently, the protein expression of STAT6 in lung tissues was decreased by mangiferin (Figure 7E). However, mangiferin did not show significant effect on STAT4 expression (data not shown).

## Discussion

Allergic asthma is one of the most common chronic respiratory diseases in children and adults. Yet it is not completely curable. It is a disease of airway inflammation that recruits various inflammatory cells, cytokines and inflammatory mediators [4]. Our previous studies have shown the anti-inflammatory activity of mangiferin in several experimental disease models [15], [29]. In the present study, we demonstrated the inhibitory effect of mangiferin on airway inflammation using a classical asthmatic model, in BALB/c mice, of sensibilization with OVA. Consistent with a report [33], both mangiferin and dexamethasone caused a significant reduction of airway inflammation around bronchi and blood vessels, inhibition of eosinophil-rich leukocytes infiltration in BALF and OVA-specific IgE levels in serum. Also, we observed the infiltrating cells, in the inflamed tissue, seemed to be leukocytes based on morphology under microscope, these cells could be the targets of mangiferin.

Asthma is often triggered by mast cells activated by IgE-mediated allergic reaction. Activated mast cells release various chemical mediators, including LTC_4_ and PGD_2_, which played an important role in late phase allergic reactions in the pathophysiology of asthma. In asthma, overproduction of PGD_2_ and LTC_4_ results in an increase in levels of Th2 cytokines and decrease in Th1 cytokines expressions, accompanied by the enhanced accumulation of eosinophils and lymphocytes in the lung [44], [45], [46]. Taking into account aforementioned reasons, we determined the effects of mangiferin on the expression of LTC_4_ and PGD_2_ in BALF, and we found that mangiferin exhibited the significant inhibition of PGD_2_ expression and a slightly reduction on the level of LTC_4_ in BALF.

To investigate the underlying immunoregulatory mechanism of mangiferin on anti-asthma, we detected serum levels of 20 cytokines/chemokines associated with airway inflammation by antibody array. Our findings showed that mangiferin markedly inhibited expressions of Th2 cytokines (including IL-4, IL-5 and IL-13) and elevated levels of Th1 cytokines (including IFN-γ, IL-12 and IL-2). Allergic asthma is primarily mediated by Th2 cells, which secrete the characteristic cytokines IL-4, IL-5, and IL-13. IL-4 plays a key role in the differentiation of Th2 cells from uncommitted Th0 cells and may be important in initial sensitization to allergens. It is also important for isotype switching of B cells from producers of IgG to producers of IgE [47]. Similar to IL-4, IL-13 mainly induces IgE secretion but does not promote Th2 cell differentiation. IL-5 is critically involved in airway eosinophilia, it regulates most aspects of eosinophil behavior, including growth, maturation, adhesion,secretion and apoptosis [40], [48]. In contrast to Th2 cells, Th1 cells have an opposite effect. IFN-γ and IL-12 are predominant cytokines produced by Th1 cells. IFN-γ and IL-12 are involved in antagonism of Th2-cell responses and IgE synthesis to restrain the progress of asthma. Accordingly, examination of levels of Th1/Th2 cytokines is an important index in the evaluation of asthma. To confirm effects of mangiferin on Th1/Th2 cytokines further, we determined the mRNA expressions of IFN-γ, IL-12, IL-4, IL-5, and IL-13 in lung tissues. As expected, the result of RT-PCR is consistent with that of antibody array. This result suggests that mangiferin could be able to regulate the immune response of Th1/Th2 cells. However, unlike mangiferin, dexamethasone suppressed Th2 (IL-4, IL-5 and IL-13) responses as well as Th1 (IFN-γ and IL-12) responses (Figure 4, 5), which is likely to increase patients' susceptibility on infection [49]. These findings suggest that mangiferin may offer clinical advantages over corticosteroids. Additionally, antibody array also demonstrated that mangiferin was able to down-regulate levels of IL-3, IL-9, IL-17, RANTES, TNF-α and up-regulate the IL-10 expression in serum, which indicates that, in addition to modulating the immune responses of Th1/Th2 cells, mangiferin possibly exerts the anti-asthmatic effect through regulation of other cytokines.

In physiological condition, Th0 cells differentiate to Th1 and Th2 cells proportionally and keep their amount in a relative dynamic balance. Whenever this balance is disturbed, diseases will occur. It has been reported that the imbalanced Th1/Th2 cells exist in patients with allergic asthma [50]. The alternation of Th1/Th2 ratio is confirmed to be an initial factor for asthma that increases airway inflammation [51]. Therefore, it could be potential beneficial remedy for asthma treatment if the imbalanced status of Th1/Th2 cells can be reversed. We tried to use intracellular cytokine staining to evaluate ratio changing of Th1 and Th2 caused by OVA, but for some reasons we did not get positive results. Considering the limited number OVA-specific CD4+ T cells via sorting process, together with a few published articles [52], [53] that support similar findings without sorting the infiltrating OVA-specific CD4+ T cells, we used total CD4+ T cells in current study. We found that mangiferin significantly reduced the frequencies of Th2 cells in a dose-dependent manner, meanwhile increased the frequencies of Th1 cells. Thus, Th1/Th2 ratio was restored to close to the normal level by mangiferin at 200 mg/kg. These findings strongly suggest that mangiferin could restore the balanced of Th1/Th2 cells in asthma.

STATs are a group of transcription factors that transmit signals from the extracellular milieu of cells to the nucleus. They are pivotal for the signaling of many cytokines that are mediators of allergic inflammation and impact various cell types critical to allergy including lymphocytes, mast cells, eosinophils, dendritic cells, and epithelial cells [54]. It have been confirmed that activation of STAT4 and STAT6 is pivotal in Th0 cell differentiate along the Th1 and Th2 pathway, respectively [5]. The STAT6-signaling pathway is mainly induced by IL-4, which induces STAT6's homodimerization and subsequent translocation into nucleus, and rapidly induces the expression of GATA-3. Expression of GATA-3 is followed by induction of the transcription factor c-MAF, a potent IL-4 gene specific activator. This forms a positive feedback loop for IL-4 induction of GATA-3 and Th2 differentiation [55], [56]. Whereas, the STAT4-signaling pathway is primarily induced by IL-12, and activation of STAT4 induces the expression of T-bet, which activates the IFN-γ gene by chromatin remodelling, leading to secretion of IFN-γ and increases expression of the IL12Rb2 chain, further enhancing IL-12 signals. [57], [58]. Dysregulation of STAT4/6 signaling has been involved in allergic asthma [7], [8], therefore, highlighting the importance of these ubiquitous molecules in allergic asthma and the potential of these pathways as a target for therapeutic intervention [59]. It remains unclear whether the protective effect of mangiferin against asthma is associated with STAT4/6-signaling pathway. To address these issues, the activity and expression of STAT4, STAT6, GATA-3 and T-bet were detected by western blot and IHC in repeatedly OVA-challenged mice. We found that the levels of p-STAT6 and GATA-3 in the lung tissues were markedly inhibited by mangiferin. Moreover, the expressions of p-STAT4 and T-bet were slightly enhanced by mangiferin. This result suggests that mangiferin mainly inhibits the STAT-6 signaling pathway and consequently modulates the imbalance of Th1/Th2 cells differentiation.

## Conclusions

In this study, we demonstrated that mangiferin inhibited OVA-induced asthmatic response by reducing airway inflammation, PGD_2_ production and OVA-specific IgE level. Mangiferin restored Th1/Th2 cytokines balance through an increase in Th1 and a decrease in Th2. It also inhibited the levels of p-STAT6 and GATA-3. Our results confirm the immunomodulatory effect of mangiferin on anti-asthma. The underlying mechanism may contribute to the modulation of Th1/Th2 imbalance via inhibiting the STAT6 signaling pathway. Together, our findings suggest that mangiferin could be used as a potential immunotherapeutic agent for patients with allergic asthma.
